# The Passing of the Doctor's Horse

**Published:** 1910-01-29

**Authors:** 


### The Hospital

A Journal of

The Medical Sciences and Hospital Administration.

New Series. No. 154, Vol. VI. [No. 1226 Vol. XLVII.] SATURDAY, JANUARY 29, 1910.

### THE PASSING OF THE DOCTOR'S HORSE.

When the celebrated conversation of Shandy *père*, my Uncle Toby, and Dr. Slop had got round to sailing chariots, the personage last-named gave it a slight turn towards the matter in hand by means of the following remark: "And I have often wondered . . . why none of our gentry, who live upon large plains like this of ours (especially they whose wives are not past child-bearing) attempt nothing of this kind; for it would not only be infinitely expeditious upon sudden calls to which the sex is subject—if the wind only served—but would be excellent good husbandry to make use of the winds, which cost nothing and which eat nothing, rather than horses, which (the devil take 'em) both cost and eat a great deal." The doughty obstetrician in question had, as we know, neither the figure nor the accomplishments of a horseman, a fact which may partly explain the dislike he thus expresses. His anticipation happens to have been realised pretty nearly; although few are found to claim any reduction in expenditure thereby, mechanical means of transport have almost done away with horses for the professional use of medical men. The causes of the change are evident. Many more visits can be paid on the car than with the trap, although this seldom means much increase in income, because unfortunately the takings of a medical practice depend upon what patients can afford, or are accustomed to think they can afford, rather than upon the amount of work done and attention shown. The most urgent case can be taken first irrespective of its convenient order in the morning round; also the saving of time has given a little leisure to practioners who formerly had none, and a night call, which to a merciful man with a small stable meant getting out his bicycle, may mean so no more—all these advantages are certain. Then, too, it is not good for one's opponent to be the only medical motorist in the neighbourhood, because patients like to have a doctor they can get quickly in time of need, and moreover are nowadays coming to expect all medical visits to follow upon a summons within the time it is possible for them to be reached at the travelling rate of from fifteen to twenty miles an hour. The progressive medical man is thus almost forced to motor, but it is a tactless question to ask him to compare the cost with former stable expenses. There is another disadvantage, however, which somehow people do not seem to mind owning to, and that is as regards personal taste, extrinsic advantages being left out of the question. If one is not dealing with a highly impatient man, or a born mechanician, or a pronounced humanitarian, pretty certainly a little regret will be expressed for the passing of the horse.

There are, of course, some who must be as prejudiced in favour of the old way of things as those just mentioned are in favour of automobilism. Happily extreme conservatism was never very common in the medical profession, and is getting to be rarer still. Less happily (for most) so are the pecuniary means which enable a man to take up expensive recreations like breeding prize hackneys, the chief employment of which is to ramp round the show-ring to thunderous cheering, "trotting over wheelbarrows." Hunting three or four days a week, too, keeping an assistant to do the work of the practice, with a mounted groom who knows the country to find the principal in case of need, cannot be done for nothing. The ordinary practitioner, however, is not a mere sportsman—or a Sir Leicester Dedlock—or half a farmer, any more than, as we have seen, he is an engineer spoilt, or a speed maniac, or a sceptic as to the justifiableness of enslaving lower animals. The enjoyment horseflesh gives him can be explained very simply. Probably in most cases—explanations are apt to be prosaic—it is just that horse-mastership is more attractive than car-mastership; it appeals to much older implanted instincts, and to much commoner ones, since Mr. Wells' mechanic type is not yet established. Settling the details of forage, bitting, shoeing, etc., the little daily strife every good groom provokes by his assumptions of professional superiority, and the look of a neat stable yard on a fine morning recreate a man much more than reckoning up depreciation of machinery in a converted coachhouse smelling of oil.

But there may be sentiment present too. Many a country doctor cherishes recollections of some wonderful old mare (seldom more than one, because good horses, like good servants of every description, are few and far between), as well known in the parish as the rector or the postman, not allowed to be touched with a whip save by the master himself, which has helped to build up the practice and proved one of its most considerable assets, and which, always willing and never sick or sorry, has done the best of service for a decade and a half—service which they requite, in the strange conventional way, by shooting her as soon as she begins to fail. And then there are the various manifestations of "horse pride," the gratification of which is conditioned by the state of the owner's finances. Lastly, there is that irrational person the born horse-lover; not a countryman by birth and upbringing, perhaps, any more than Jorrocks was, but having an abiding interest in horses as firmly rooted in him as that worthy had the love of fox-hunting. This is the individual who will astonish a visitor by jumping up from the table and running to the window to look at a passing string of cart horses because one of them "walks well." Medical training does nothing to impair this taste of his, and for him, needless to say, the lines will have fallen in pleasant places when his occupation brings him into contact with the horse.

